# Detection of 7-Dehydrocholesterol and Vitamin D3 Derivatives in Honey

**DOI:** 10.3390/molecules25112583

**Published:** 2020-06-02

**Authors:** Tae-Kang Kim, Venkatram Atigadda, Pawel Brzeminski, Adrian Fabisiak, Edith K. Y. Tang, Robert C. Tuckey, Andrzej T. Slominski

**Affiliations:** 1 Department of Dermatology, University of Alabama at Birmingham, Birmingham, AL 35294, USA; tkim@uabmc.edu (T.-K.K.); venkatra@uab.edu (V.A.); pbrzeminski@chem.uw.edu.pl (P.B.); afabisiak@chem.uw.edu.pl (A.F.); 2 VA Medical Center, Birmingham, Birmingham, AL 35294, USA; 3 Department of Chemistry, University of Warsaw, Pasteura 1, 02-093 Warsaw, Poland; 4 School of Molecular Sciences, The University of Western Australia, Perth, WA 6009, Australia; edith.tang@uwa.edu.au (E.K.Y.T.); robert.tuckey@uwa.edu.au (R.C.T.)

**Keywords:** honey, bees, vitamin D3, 7-dehydrocholesterol, lumisterol3, hydroxyderivatives of vitamin D3

## Abstract

20(*S*)-Hydroxyvitamin D3 (20(OH)D3) is an endogenous metabolite produced by the action of CYP11A1 on the side chain of vitamin D3 (D3). 20(OH)D3 can be further hydroxylated by CYP11A1, CYP27A1, CYP24A1 and/or CYP27B1 to several hydroxyderivatives. CYP11A1 also hydroxylates D3 to 22-monohydroxyvitamin D3 (22(OH)D3), which is detectable in the epidermis. 20-Hydroxy-7-dehydrocholesterol (20(OH)-7DHC) has been detected in the human epidermis and can be phototransformed into 20(OH)D3 following the absorption of ultraviolet B (UVB) energy by the B-ring. 20(OH)D3 and its hydroxyderivatives have anti-inflammatory, pro-differentiation and anti-proliferative effects, comparable to 1,25-dihydroxyvitamin D3 (1,25(OH)_2_D3). Since cytochromes P450 with 20- or 25-hydroxylase activity are found in insects participating in ecdysone synthesis from 7-dehydrocholesterol (7DHC), we tested whether D3-hydroxyderivatives are present in honey, implying their production in bees. Honey was collected during summer in the Birmingham area of Alabama or purchased commercially and extracted and analyzed using LC-MS. We detected a clear peak of *m*/*z* = 423.324 [M + Na]^+^ for 20(OH)D3 corresponding to a concentration in honey of 256 ng/g. We also detected peaks of *m*/*z* = 383.331 [M + H − H_2_O]^+^ for 20(OH)-7DHC and 25(OH)D3 with retention times corresponding to the standards. We further detected species with *m/z* = 407.329 [M + Na]^+^ corresponding to the RT of 7DHC, D3 and lumisterol3 (L3). Similarly, peaks with *m/z* = 399.326 [M + H − H_2_O]^+^ were detected at the RT of 1,25(OH)_2_D3 and 1,20-dihydroxyvitamin D3 (1,20(OH)_2_D3). Species corresponding to 20-monohydroxylumisterol3 (20(OH)L3), 22-monohydroxyvitamin D3 (22(OH)D3), 20,23-dihydroxyvitamin D3 (20,23(OH)_2_D3), 20,24/25/26-dihydroxyvitamin D3 (20,24/25/26(OH)_2_D3) and 1,20,23/24/25/26-trihydroxyvitamin D3 (1,20,23/24/25/26(OH)_3_D3) were not detectable above the background. In conclusion, the presence of 7DHC and D3 and of species corresponding to 20(OH)-7DHC, 20(OH)D3, 1,20(OH)_2_D3, 25(OH)D3 and 1,25(OH)_2_D3 in honey implies their production in bees, although the precise biochemistry and photochemistry of these processes remain to be defined.

## 1. Introduction

Photochemical transformation of 7DHC to D3 after absorption of ultraviolet B (UVB; λ = 280–320 nm) represents a fundamental reaction for the biology of vertebrates [1,2,3]. The canonical pathway of D3 activation (D3→25(OH)D3→1,25(OH)_2_D3) that occurs in liver and kidney involves its sequential hydroxylation at C25 by CYP2R1 or CYP27A1 and at C1α by CYP27B1, producing the hormonally active 1,25(OH)_2_D3 [2,4,5,6,7]. D3 also undergoes the same hydroxylation sequence in cells of peripheral tissues, especially skin [4,8,9]. 1,25(OH)_2_D3 not only regulates calcium homeostasis but also has pleiotropic activities, which include the stimulation of differentiation and the inhibition of proliferation of cells of different lineages, acting as an anti-cancerogen [2,8,10,11,12,13,14,15,16]. It also inhibits adaptive immunity and inflammation and stimulates the epidermal barrier and hair growth. In addition, 1,25(OH)_2_D3 acts as a chemopreventive agent in proliferative, malignant and inflammatory cutaneous disorders [2,8,10,11,12,13,14,15,17,18]. It is believed that the majority of these functions are mediated via interaction with the vitamin D receptor (VDR), a member of the superfamily of nuclear hormone receptors, which binds to the VDR responsive element (VDRE) to influence expression of responsive genes [8,12,19].

Over the last two decades, evidence has accumulated for alternative, non-canonical pathways of vitamin D activation initiated by CYP11A1, an obligatory enzyme of steroidogenesis [7,20,21,22]. Specifically, CYP11A1 hydroxylates D3 at C20 to produce 20(OH)D3, which it further metabolizes to 20,22(OH)_2_D3 [23,24,25], 20,23(OH)_2_D3 and 17,20,23-trihydroxyvitamin D3 (17,20,23(OH)_3_D3) [26]. Similarly, the vitamin D2 side chain is also hydroxylated by CYP11A1 to produce 20-monohydroxyvitamin D2 (20(OH)D2) as the major metabolite [27,28]. 20(OH)D3/D2 and 20,23(OH)_2_D3 can be further hydroxylated at C1α by CYP27B1 [29,30]. Many of the CYP11A1 hydroxymetabolites are produced in vivo [31,32,33]. They exert similar phenotypic effects to those of 1,25(OH)_2_D3 [20], however mediating their actions not only through the VDR [34,35] but also through retinoic acid orphan receptors (ROR) [36,37] and the aryl hydrocarbon receptor (AhR) [38]. Of great interest is that the novel CYP11A1-derived hydroxyderivatives of D3 show photoprotective activities against UVB [39,40,41] which are similar to those exerted by 1,25(OH)_2_D3 [42,43,44].

Similar to animals, plants are also capable of producing D3 from 7DHC [45,46,47,48,49]. In addition, some plants are also capable of further oxidizing D3 to produce 25(OH)D3 and 1,25(OH)_2_D3 [45,46,47,49,50]. In the case of insects, recent reports demonstrate that D3 is also produced in these organisms after exposure to UVB [51]. It is also well established that multiple species of insects and crustaceans synthesize 7DHC from dietary sterols [52,53,54] and further convert it to vitamin D3 after exposure to UVB. In addition, both 20-hydroxylase and 25-hydroxylase enzymes, required for the production of 20-hydroxyecdysone from 7DHC, are found in insects [55,56]. In this report, we investigated whether D3 and its hydroxyderivatives are present in honey, thus implicating honeybees in the production of secosteroids. 

## 2. Results 

**Figure 1:**
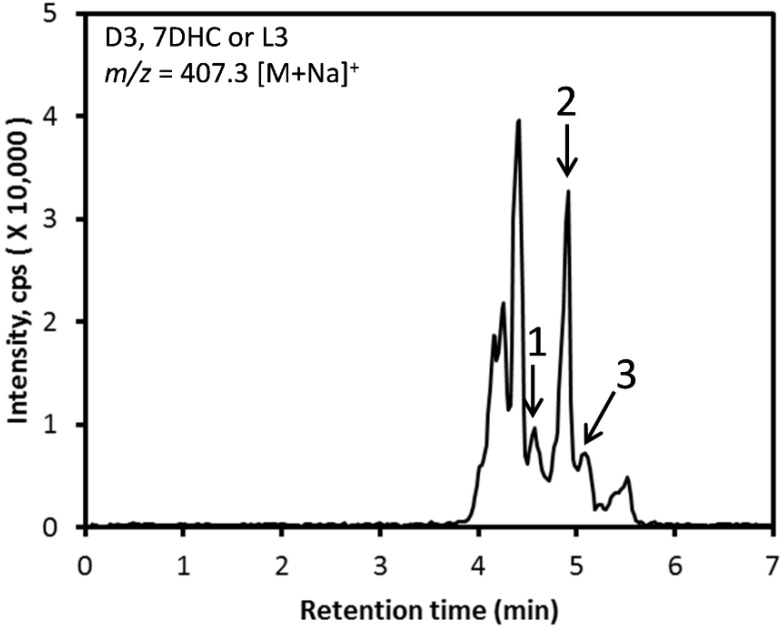
Detection of D3, 7DHC and L3 in honey. Whole honey extract was analyzed directly using a Zorbax Eclipse Plus C18 column connected to a Xevo G2 XS equipped with an ACQUITY UPLC I-Class System (Waters, Milford, MA) using a methanol gradient. The extracted ion chromatogram (EIC) was obtained using *m/z* = 407.329 [M + Na]^+^. Arrow 1, RT of D3 standard; arrow 2, RT of 7DHC standard; arrow 3, RT of L3 standard.

**Figure 2:**
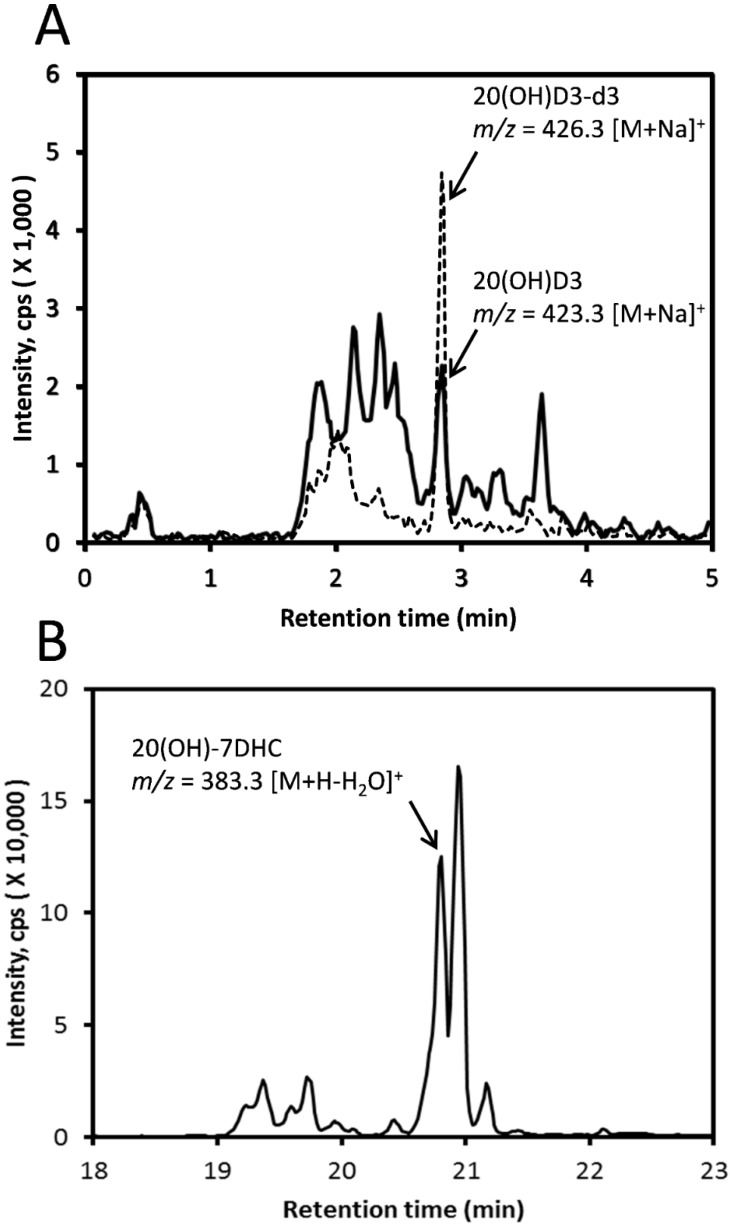
Detection of 20(OH)D3 and 20(OH)-7DHC in honey. (**A**) After a pre-purification step using an Atlantis C18 column with a methanol gradient, then analyzed using LC-MS with an ACQUITY UPLC BEH C18 column with a methanol gradient. The EIC was obtained using *m/z* = 423.324 [M + Na]^+^. (**B**) After extraction of commercially sourced honey, the sample was directly analyzed using LC-MS with a Pursuit 200Å PFP column with a methanol gradient. The EIC was obtained using *m/z* = 383.331 [M + H − H_2_O]^+^.

Honey collected during summer in the Birmingham, Alabama area or purchased commercially (see Section 4.2) was extracted into methylene chloride (CH_2_Cl_2_) and analyzed using LC-MS. Three peaks (indicated by arrows 1, 2 and 3) with *m/z* = 407.329 [M + Na]^+^ were detected (Figure 1). Based on retention time (RT) for standard samples, these peaks correspond to D3, 7DHC and L3 standards, respectively. Vitamin D species in the commercial honey extract were purified using an Atlantis C18 column (100 × 4.6 mm, 5 μm). Figure S1A demonstrates that 20(OH)D3 separates from other monohydroxyvitamin D3 derivatives including 25(OH)D3 in this system with an acetonitrile gradient, as well as with a methanol gradient (Figure S1A, inset). Epi-25(OH)D3 has identical RT as 25(OH)D3 under these chromatography conditions (not shown). Subsequent analysis of the collected samples using LC-MS with an ACQUITY UPLC BEH C18 column revealed a peak of *m/z* = 423.324 [M + Na]^+^ with a RT corresponding to standard 20(OH)D3 (Figure 2A). This major peak was chosen for quantitation. 20(OH)D3-d3 was added to the purified sample enabling the endogenous 20(OH)D3 to be measured from the ratio of 20(OH)D3-d0/20(OH)D3-d3 using a standard curve (Figure S2). The standard curve was linear over the range used (0.1 ng to 5 ng) with a slope of 12.77 ± 1.81 (*n* = 2) and coefficient of variation of r^2^ ≥ 0.998. The limit of quantification (LOQ) was 0.1 ng for which the S/N (signal to noise) ratio was 20.84 ± 2.98 (*n* = 2), and this represents the lowest point on the standard curve (Figure S3). Using this procedure, we determined that the concentration of 20(OH)D3 in commercially obtained honey is 256 ± 24 ng/g of honey (*n* = 2 by technical replicates) after correcting for all dilutions. 

20(OH)-7DHC, a potential precursor of 20(OH)D3, was detected in the extracts from honey purchased commercially. This extract was analyzed using LC-MS with a Pursuit 200Å PFP column (4.6 × 150 mm, 5 µm) for separation. The resulting chromatogram from the commercial honey gave a peak with *m/z* = 383.331 [M + H − H_2_O]^+^ with a RT corresponding to the 20(OH)-7DHC standard (Figure 2B) and another major peak which remains unidentified. 

**Figure 3:**
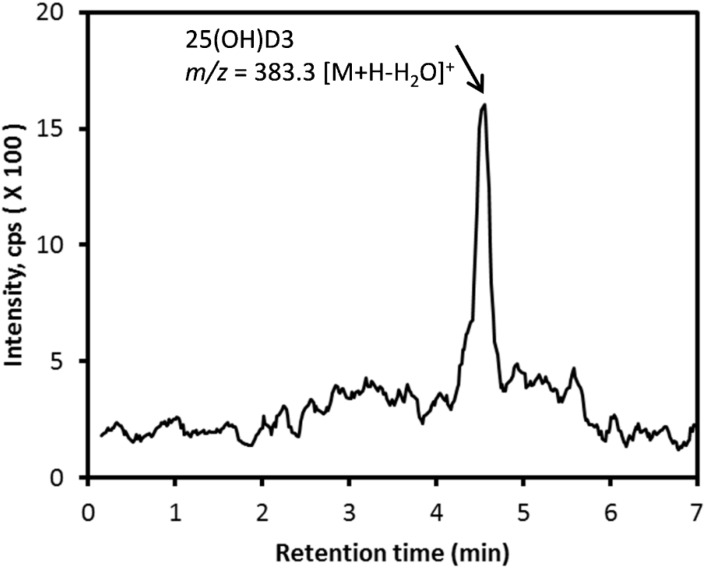
Detection of 25(OH)D3 in honey. After a pre-purification step using a C18 column with an acetonitrile gradient, the fraction was analyzed using LC-MS with a Zorbax Eclipse Plus C18 column with an acetonitrile gradient. The EIC was obtained using *m/z* = 383.331 [M + H − H_2_O]^+^.

**Figure 4:**
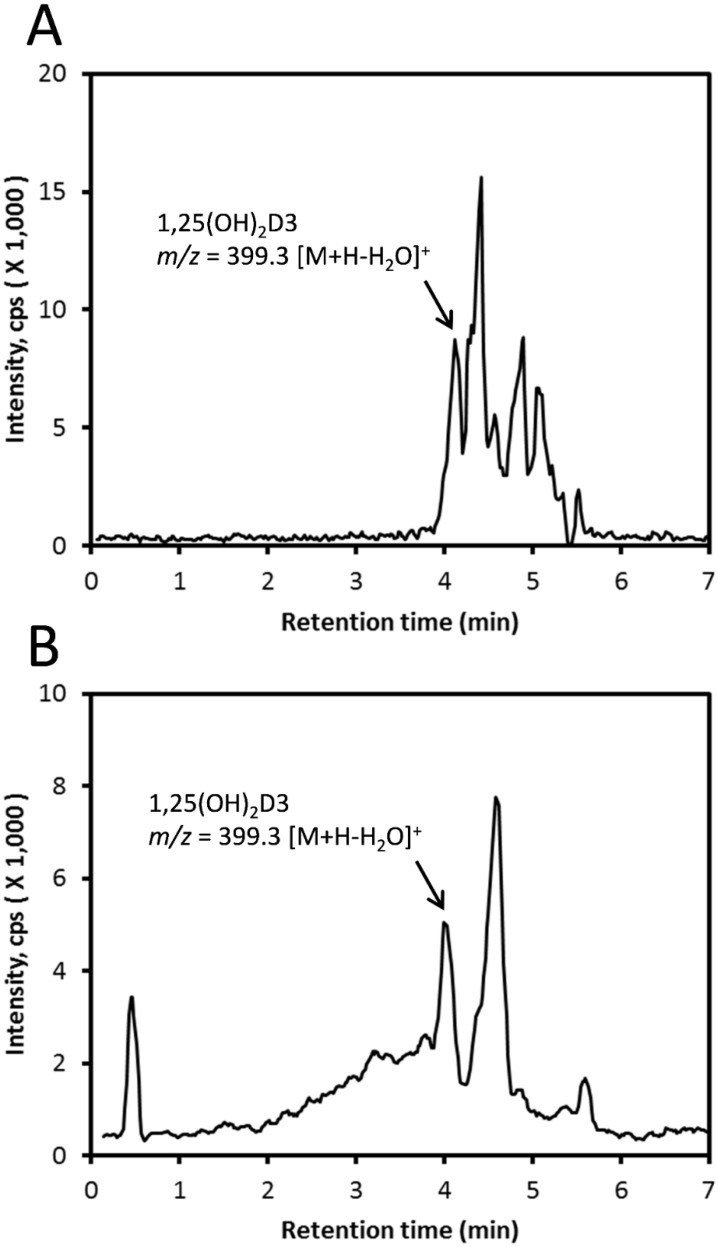
Detection of 1,25(OH)_2_D3 in honey. (**A**) Whole extract was analyzed directly using LC-MS with a Zorbax Eclipse Plus C18 column with a methanol gradient. The EIC was obtained using *m/z* = 399.326 [M + H − H_2_O]^+^. (**B**) The sample was pre-purified using a C18 column, then analyzed using LC-MS with a Zorbax Eclipse Plus C18 column with an acetonitrile gradient. The EIC was obtained using *m/z* = 399.326 [M + H − H_2_O]^+^.

Any 25(OH)D3 in the honey was also pre-purified (prior to LC-MS) using HPLC with a C18 column (250 × 4.6 mm, 5 μm particle size) with the fraction having the same RT as standard 25(OH)D3 being collected (see Figure S1A for typical separation). This fraction was then analyzed using LC-MS with a Zorbax Eclipse Plus C18 column (2.1 × 50 mm, 1.8 μm) with an acetonitrile gradient resulting in a peak with *m/z* = 383.331 [M + H − H_2_O]^+^ that had a RT matching standard 25(OH)D3 (Figure 3). Using the same extract but without pre-purification, we detected a peak with *m/z* = 399.326 [M + H − H_2_O]^+^ corresponding to the RT of standard 1,25(OH)_2_D3 (Figure 4A). The same mass peak was detected in extracts of commercial honey after pre-purification on a C18 column (as in Figure S1B) followed by LC-MS using a Zorbax Eclipse Plus C18 column with an acetonitrile gradient (Figure 4B).

**Figure 5:**
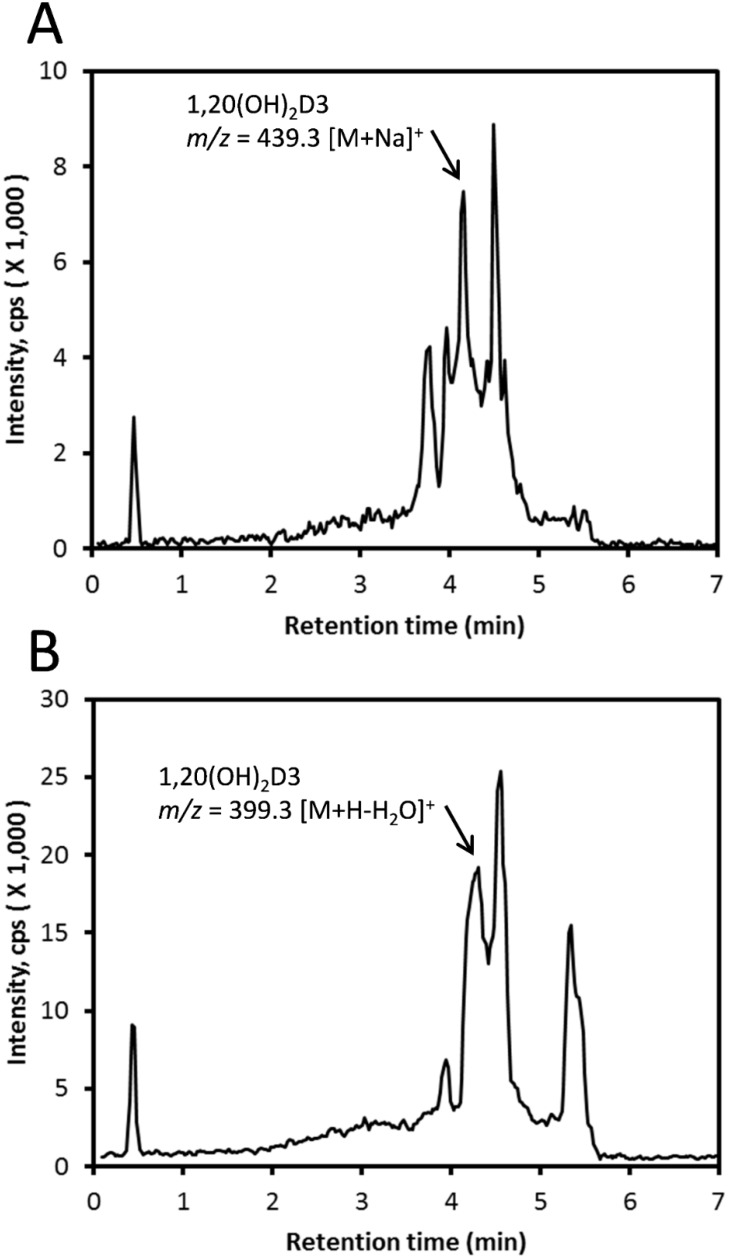
Detection of 1,20(OH)_2_D3 in honey. (**A**) The peak corresponding in RT to standard was collected from commercially available honey using an Atlantis C18 column using a methanol gradient, then analyzed using LC-MS with an ACQUITY UPLC BEH C18 column with a methanol gradient. The EIC was obtained using *m/z* = 439.319 [M + Na]^+^. (**B**) The local honey sample was pre-purified using a C18 column, then analyzed using LC-MS with a Zorbax Eclipse Plus C18 column with an acetonitrile gradient. The EIC was obtained using *m/z* = 399.326 [M + H − H_2_O]^+^.

Any 1,20(OH)_2_D3 in the commercially available honey was pre-purified on an Atlantis C18 column (100 × 4.6 mm, 5 μm) using a gradient of methanol and water, as described in Materials and Methods, and then analyzed using LC-MS with an ACQUITY UPLC BEH C18 column (2.1 × 50 mm, 1.7 μm) with a methanol gradient. 1,20(OH)_2_D3 was detected with *m/z* = 439.319 [M + Na]^+^ (Figure 5A). In addition, the local honey extract was similarly pre-purified then analyzed using LC-MC with a Zorbax Eclipse Plus C18 column. The EIC showed a peak with *m*/*z* = 399.326 [M + H − H_2_O]^+^ with a RT corresponding to the 1,20(OH)_2_D3 standard (Figure 5B).

Table 1 shows a summary of the information on the metabolites detected in honey, including the LC system used, RT and detected masses. Additional analysis of honey extracts showed that species corresponding to 20(OH)L3, 22(OH)D3, 20,23(OH)_2_D3, 20,24/25/26(OH)_2_D3, 24,25(OH)_2_D3 and 1,2,23/24/25/26(OH)_3_D3 were not detectable in the honey extracts using LC-MS (data not shown).

## 3. Discussion

Secosteroids play important roles in normal cellular functions including maintaining calcium and phosphorus homeostasis, stimulating cell differentiation and inhibiting proliferation. Consumption or supplementation of foods with some of these secosteroids from natural sources may provide health benefits. Previous reports indicate that 7DHC, a precursor for the synthesis of the steroidal prohormone, ecdysone, is present in insects [53,54,57]. In addition, vitamin D3, which is a 7DHC photoproduct resulting from exposure to UVB, is present in many insects as well [51]. Therefore, we investigated whether any of these secosteroids and/or their hydroxylated metabolites were present in natural honey. Using LC-MS, we identified 7DHC, D3 and L3 in natural unprocessed honey. The presence of L3 along with D3 was expected since exposure to UVB, depending on the dose, can transform pre-D3 into L3 [1,2,3]. 

In addition to the above three natural metabolites, we also detected 20(OH)-7DHC, 20(OH)D3, 1,20(OH)_2_D3, 25(OH)D3 and 1,25(OH)_2_D3 based on their RT and mass. Detection of these hydroxy-metabolites was unexpected but can be rationalized by the presence of both 20-hydroxylase and 25-hydroxylase activities in insects that are involved in 20-hydroxyecdysone synthesis from 7-dehydrocholesterol [55,56]. Alternatively, it is well documented that some plant species can produce 25(OH)D3 and 1,25(OH)_2_D3 [45,46,47,49,50], which could possibly be a source of 25(OH)D3 and 1,25(OH)_2_D3 in honey. 

Using an LC-MS assay utilizing 20(OH)D3-d3 as internal standard, we determined the concentration of 20(OH)D3 in honey to be 0.26 µg/g, corresponding to approximately 10 IU/g based on the conversion for vitamin D3 (400 IU = 10 µg). While this may seem relatively low, it must be noted that 20(OH)D3, unlike vitamin D3, is an activated form of the vitamin. It is non-calcemic but exerts many of the other activities of 1,25(OH)_2_D3 seen in cell culture and in animal studies, with comparable potency [20,39,44,58,59,60]. Thus, while the amount of 20(OH)D3 in honey is relatively low, it could potentially be of physiological significance.

**Scheme 1:**
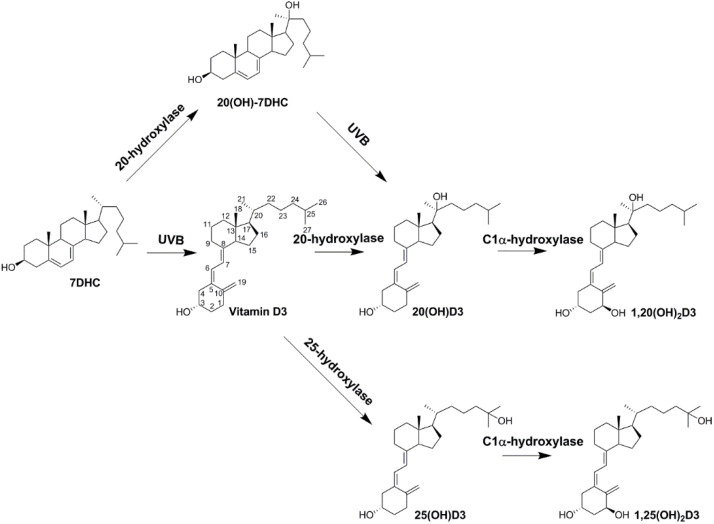
Proposed scheme for the metabolism of D3 and 7DHC leading to the appearance of their hydroxyproducts in honey.

In the mammalian system, 20(OH)D3 is produced through the action of CYP11A1 on D3 and is detectable in human skin [21,33]. 20(OH)-7DHC is also detectable in human skin; however, it is unclear which enzyme is responsible for its production [61]. In mammals, both CYP11A1 and CYP27A1 can act on substrates (including 7DHC) with an intact B-ring as well as on vitamin D derivatives (secosteroids) with a broken B-ring [7,21]. If the 20/25-hydroxylases involved in ecdysone synthesis also have this ability, they may be responsible for the production of the hydroxyderivatives of both 7DHC and D3 detected in honey. The photochemical transformation of the corresponding 7DHC hydroxyderivatives, especially for 20(OH)-7DHC, may also be responsible for the hydroxyvitamin D3 species observed in honey, without the requirement for direct hydroxylase activity on D3. A proposed scheme for production of the detected metabolites is shown in Scheme 1. The significance of the production of these compounds in bees remains to be investigated. However, it is noteworthy that in certain plants vitamin D hydroxyderivatives promote differentiation and elongation of roots, possibly via the stimulation of calcium uptake [46,49]. We also speculate that they may have photoprotective functions, as described in the human skin [39,40,41,42,43,44].

In conclusion, we are reporting the presence of 7DHC, D3 and L3, and of species corresponding to 20(OH)-7DHC, 20(OH)D3, 1,20(OH)_2_D3, 25(OH)D3 and 1,25(OH)_2_D3 in honey, which implies their production in bees.

## 4. Materials and Methods

### 4.1. Chemicals

HPLC grade methylene chloride from Fisher Scientific (Hampton, NH, USA) was used for honey extraction. For HPLC or LC-MS, LC-MS grade acetonitrile, water and formic acid were purchased from Fisher Scientific (Hampton, NH, USA), and LC-MS grade methanol was purchased from Honeywell international Inc. (Charlotte, NC, USA). 20(OH)D3, 22(OH)D3 and 1,20(OH)_2_D3 were synthesized enzymatically using recombinant bovine CYP11A1, as previously described [24,26,62]. 20(OH)D3-d3 was similarly synthesized from deuterated vitamin D3 (6, 19, 19-d3) (Sigma Aldrich, St. Louis, MO, USA). 20(OH)-7DHC and 20(OH)L3 were synthesized chemically, as described previously [63]. Other CYP11A1-derived hydroxyderivatives of vitamin D3 were synthesized as described previously [64,65,66,67,68,69]. The identities of all synthesized secosteroidal standards were determined using mass spectrometry and NMR at the time of synthesis [24,26,62,63,64,65,66,67,68,69]. 25(OH)D3 and 1,25(OH)_2_D3 were purchased from Sigma Aldrich (St. Louis, MO, USA). Vitamin D3 and 7DHC were purchased from Honeywell international Inc. (Charlotte, NC, USA). Lumisterol3 was purchased from Toronto Research Chemicals, Inc. (North York, ON, Canada).

### 4.2. Source of Honey, Extraction and Pre-Purification Protocols

Honey (70 mL) collected during the summer months in the Birmingham, Alabama, area was extracted with methylene chloride (175 mL). The organic fraction was aliquoted and dried under N_2_ gas. These samples were defined as honey extract from the local source. In addition, commercially available unprocessed honey from Nature Nate’s Corporate (McKinney, TX, USA) was purchased from Walmart. The honey (100 g) was dissolved in distilled water (350 mL) and transferred to a 1 L extraction funnel. The aqueous layer was extracted with CH_2_Cl_2_ (3 × 150 mL). The combined organic layers were washed with distilled water (3 × 100 mL), brine (2 × 100 mL) and dried over sodium sulfate. The solution was then filtered using a sintered glass Buchner funnel and the solvent evaporated using a rotary evaporator while maintaining the water bath temperature below 35 °C to give a semisolid (200 mg). These samples were defined as honey extract from the commercial source.

### 4.3. Liquid Chromatography-Mass Spectrometry (LC-MS)

Extracted samples from the local source were redissolved in 10 mL methanol and aliquots analyzed with a qTof LC-MS using Xevo G2 XS equipped with an ACQUITY UPLC I-Class System (Waters, Milford, MA, USA), or analyzed after pre-purification carried out using a 1260 Infinity II HPLC system with a C18 column (250 × 4.6 mm, 5 μm particle size) (Waters, Milford, MA, USA). The pre-purification was performed using conditions as follows: 0–15 min, 40–100% acetonitrile in water (*v*/*v*) at a flow rate of 0.5 mL/min; 15–45 min, 100% acetonitrile at a flow rate of 0.5 mL/min; 45–65 min, 100% acetonitrile at a flow rate of 1.5 mL/min. All standard samples were well separated from each other as indicated by RT (Figure S1). Chromatography for LC-MS was performed using a Zorbax Eclipse Plus C18 column (2.1 × 50 mm, 1.8 μm) (Agilent Technology, Santa Clara, CA, USA), an ACQUITY UPLC BEH C18 column (2.1 × 50 mm, 1.7 μm), an Atlantis C18 column (100 × 4.6 mm, 5 μm) (Waters, Milford, MA, USA) or a Pursuit 200Å PFP column (4.6 × 150 mm, 5 µm) (Agilent Technology, Santa Clara, CA, USA). For the Zorbax Eclipse Plus C18 column or the ACQUITY UPLC BEH C18 column (1,20(OH)_2_D3 analysis), elution was performed with a gradient of methanol or acetonitrile in water (all containing 0.1% formic acid) as follows: 0–3 min, 20–60% methanol or acetonitrile (*v*/*v*); 3–3.2 min, 60–97% methanol or acetonitrile in water (*v*/*v*); 3.2–4.8 min, 97% methanol or acetonitrile in water (*v*/*v*) at a flow rate of 0.3 mL/min. For the ACQUITY UPLC BEH C18 column, elution was performed with a gradient of methanol in water (all containing 0.1% formic acid) as follows: 0–2 min, 40% methanol in water (*v*/*v*); 2–3 min, 40–85% methanol in water; 3–6 min, 97% methanol in water (*v*/*v*) at a flow rate of 0.3 mL/min. For the Atlantis C18 column, elution was with a gradient of methanol in water (containing 0.1% formic acid for LC-MS) as follows: 0–20 min, 85–100% methanol in water; 20–30 min, 100% methanol in water (*v*/*v*) at a flow rate of 0.5 mL/mL. For the Pursuit 200Å PFP column, elution was performed with a gradient of methanol (containing 0.1% formic acid) as follows: 0–20 min, 40–100% methanol in water (*v*/*v*); 20–30 min, 100% methanol in water (*v*/*v*) at a flow rate of 0.5 mL/mL. All injections were performed after re-equilibration with the initial solvent until the delta column pressure reached around 10 psi. Masses were scanned from 100 to 1000 Da in the positive mode using the continuum mode with a scan time of 1 s. The capillary voltage was 1.7 kV with 40 V as the cone voltage. The desolvation gas flow rate was 800 L/h with a source temperature of 120 °C. Leucine enkephalin (200 ng/mL, *m*/*z* = 556.2771) was used as the lockspray reference compound at a flow rate of 10 µL/min with the lockspray interval being 10 s and a scan time of 1 s. EICs were obtained using *m*/*z* = 407.329 [M + Na]^+^ for D3 and 7DHC, 383.331 [M + H − H_2_O]^+^ or 423.324 [M + Na]^+^ for 25(OH)D3, 20(OH)D3 and 20(OH)-7DHC, 399.326 [M + H − H_2_O]^+^ for 1,25(OH)_2_D3 and 1,20(OH)_2_D3, using Waters MassLynx 4.1 software (Waters, Milford, MA, USA).

## Figures and Tables

**Table 1:** Summary of LC conditions used for detected metabolites with RT and observed masses.

Compounds	Column ^1^	Mobile Phase ^2^	RT (min)	Detected Mass (*m*/*z*)
D3	Zorbax Eclipse Plus C18	Methanol	4.55	407.329 [M + Na]^+^
7DHC	Zorbax Eclipse Plus C18	Methanol	4.90	407.329 [M + Na]^+^
L3	Zorbax Eclipse Plus C18	Methanol	5.07	407.329 [M + Na]^+^
20(OH)D3	ACQUITY UPLC BEH C18	Methanol	2.85	423.324 [M + Na]^+^
20(OH)D3-d3	ACQUITY UPLC BEH C18	Methanol	2.85	426.340 [M + Na]^+^
20(OH)-7DHC	Pursuit 200Å PFP	Methanol	20.73	383.331 [M + H − H_2_O]^+^
25(OH)D3	Zorbax Eclipse Plus C18	Acetonitrile	4.46	383.331 [M + H − H_2_O]^+^
1,25(OH)_2_D3	Zorbax Eclipse Plus C18	Methanol	4.12	399.326 [M + H − H_2_O]^+^
	Zorbax Eclipse Plus C18	Acetonitrile	3.96	399.326 [M + H − H_2_O]^+^
1,20(OH)_2_D3	ACQUITY UPLC BEH C18	Methanol	4.14	439.319 [M + Na]^+^
	Zorbax Eclipse Plus C18	Acetonitrile	4.24	399.326 [M + H − H_2_O]^+^

^1^ Details with column sizes are described in Materials and Methods. ^2^ LC gradients (in water) and flow rates are described in Materials and Methods.

## Supplementary Materials

The following are available online, Figures S1–S3.
