# Supplementary material for: Detection of 7-Dehydrocholesterol and Vitamin D3 Derivatives in Honey
Source: Molecules. 2020 Jun 2;25(11):2583. doi: 10.3390/molecules25112583 (PMC7321140; doi:10.3390/molecules25112583)
Supplement: Supplementary file 1 [file molecules-25-02583-s001.pdf]

Supplementary Materials

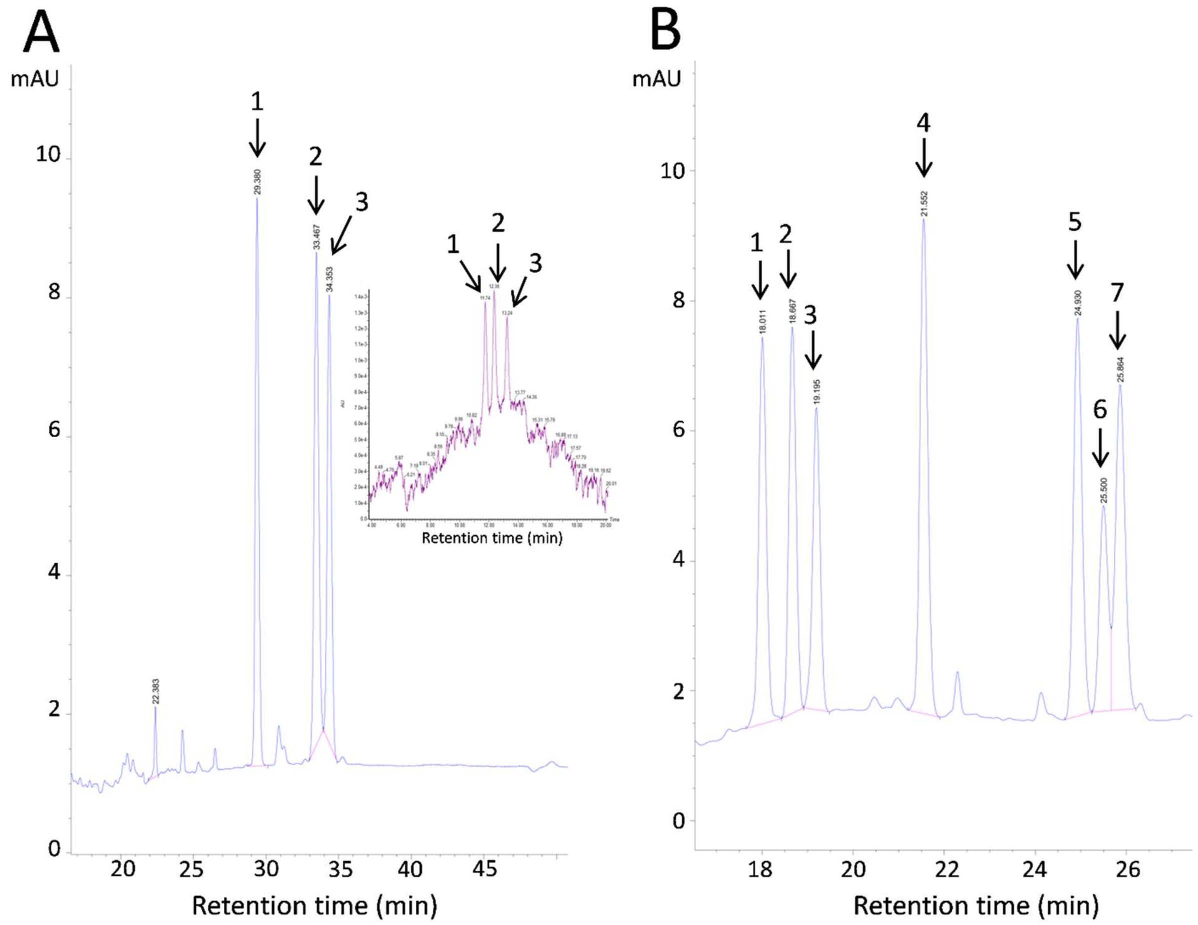

Figure S1. Chromatograms showing the separation of different monohydroxyvitamin D3 species and dihydroxyvitamin D3 species. A, separation of monohydroxyvitamin D3 species using a C18 column (250 × 4.6 mm, 5 µm particle size) with a gradient of acetonitrile. The separation shown in the inset in A was done with an Atlantis C18 column (100×4.6 mm, 5 µm) with a methanol gradient. Arrow 1: 22(OH)D3; arrow 2: 25(OH)D3; arrow 3: 20(OH)D3. B, separation of dihydroxyvitamin D3 species on an a C18 column (250 × 4.6 mm, 5 µm particle size) with a gradient of acetonitrile as described in materials and methods. Arrow 1: 20,25$(OH)_2$D3; Arrow 2: 20,26$(OH)_2$D3; arrow 3: 20,24$(OH)_2$D3; arrow 4: 1,25$(OH)_2$D3; arrow 5: 20,22$(OH)_2$D3; arrow 6: 1,20$(OH)_2$D3; arrow 7: 20,23$(OH)_2$D3.

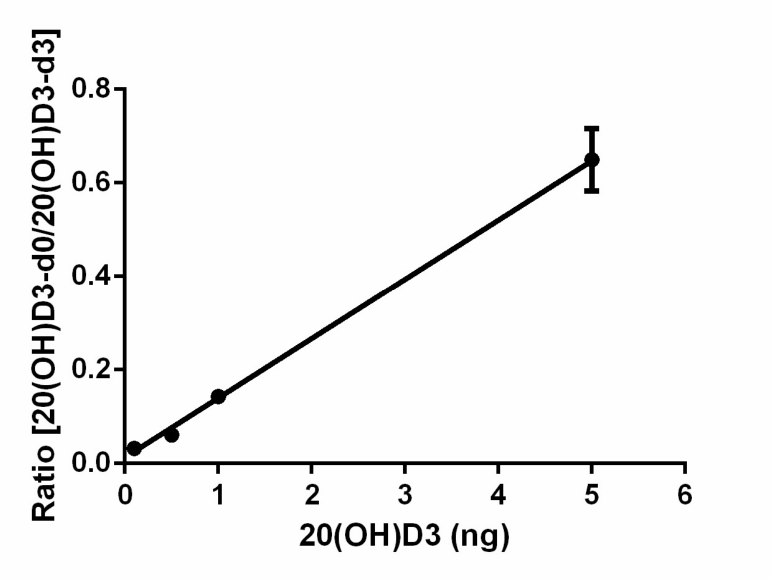

Figure S2. Standard curve for 20(OH)D3 quantification in which the peak area ratios for 20(OH)D3-d0/20(OH)D3-d3 were plotted over the range of 0.1 to 5 ng using *m/z* = 423.324 for 20(OH)D3-d0 and 426.339 for 20(OH)D3-d3. The peak area was calculated using Waters MassLynx™ Software.

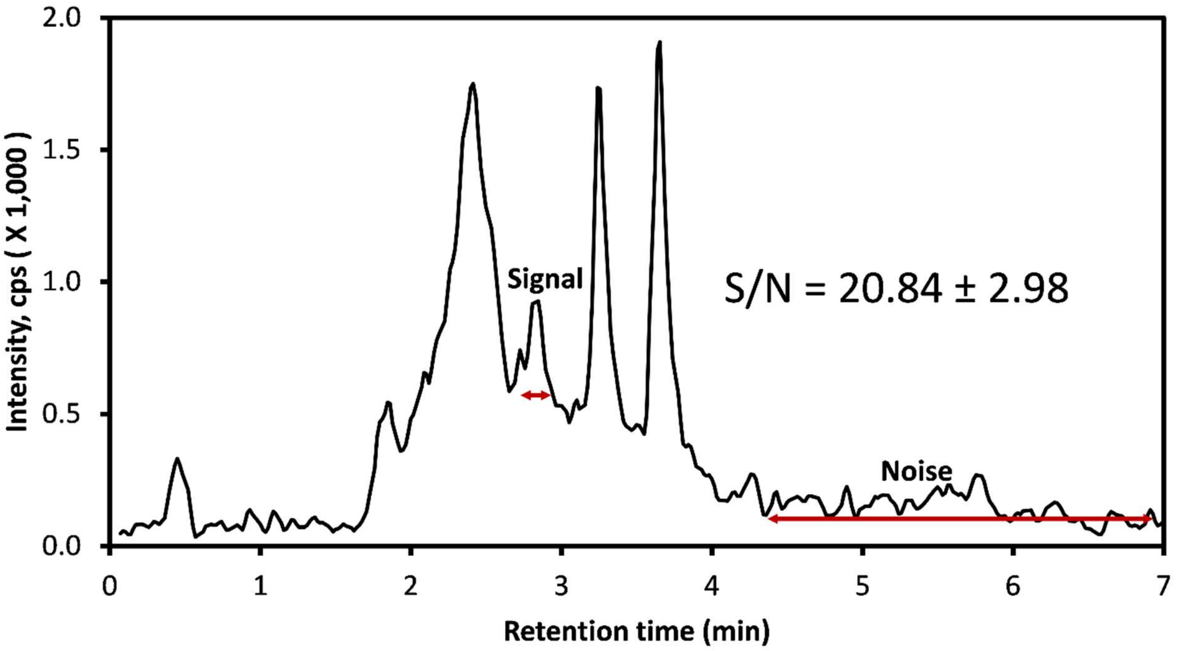

Figure S3. The limit of quantification (LOQ) was determined by calculating the ratio of signal/noise (S/N) using Waters MassLynx™ Software with a chromatogram in which 0.1 ng 20(OH)D3 was injected into an ACQUITY UPLC BEH C18 column (2.1 × 50 mm, 1.7 μm).
